# Discrimination of Aroma Characteristics for Cubeb Berries by Sensomics Approach with Chemometrics

**DOI:** 10.3390/molecules23071627

**Published:** 2018-07-04

**Authors:** Huan Cheng, Jianle Chen, Peter J. Watkins, Shiguo Chen, Dan Wu, Donghong Liu, Xingqian Ye

**Affiliations:** 1 College of Biosystems Engineering and Food Science, Zhejiang University, Hangzhou 310058, China; huancheng@zju.edu.cn (H.C.); 3090100118@zju.edu.cn (J.C.); chenshiguo210@163.com (S.C.); wudan2008@zju.edu.cn (D.W.); dhliu@zju.edu.cn (D.L.); 2 National-Local Joint Engineering Laboratory of Intelligent Food Technology and Equipment, Fuli Institute of Food Science, Zhejiang Key Laboratory for Agro-Food Processing, Zhejiang Engineering Laboratory of Food Technology and Equipment, Hangzhou 310058, China; 3 CSIRO Agriculture and Food Nutrition Unit, 671 Sneydes Road, Werribee 3030, Australia; Peter.Watkins@csiro.au

**Keywords:** cubeb berry, principal component analysis (PCA), solid-phase microextraction (SPME), hydro-distillation (HD), simultaneous distillation/extraction (SDE), gas chromatography-mass spectrometry-olfactometry (GC-MS-O)

## Abstract

The dried cubeb berries are widely used as medicinal herb and spicy condiment with special flavor. However, there is a significant definition discrepancy for cubeb berries. In this study, an efficient analytical method to characterize and discriminate two popular cubeb fruits (*Litsea cubeba* and *Piper cubeba*) was established. The aroma profiles of cubeb berries were evaluated by different extraction methods including hydro-distillation, simultaneous distillation/extraction, and solid-phase micro-extraction followed by gas chromatography-mass spectrometry-olfactometry (GC-MS-O). In total, 90 volatile compounds were identified by HD, SDE, and SPME combined with GC-MS. Principal component analysis was further applied and discriminated ambiguous cubeb berries by their unique aromas: *Litsea cubeba* was characterized by higher level of d-limonene (“fruit, citrus”), citral (“fruit, lemon”) and dodecanoic acid; α-cubebene (“herb”) was identified as a marker compound for *Piper cubeba* with higher camphor (“camphoraceous”), and linalool (“flower”). Flavor fingerprint combined with PCA could be applied as a promising method for identification of cubeb fruits and quality control for food and medicinal industries.

## 1. Introduction

*Litsea cubeba* (Lour.) Pers.(Lauraceae) gives off an aromatic odor and smells similar to an intensely lemonlike, spicy aroma. *Litsea cubeba* (*L. cubeba*) is a promising industrial crop as its fruit is rich in valuable essential oil. Recently, many reports have demonstrated the bioactivities of essential oil in *L. cubeba* [1,2,3,4]. *L. cubeba* has been widely employed in a flavoring or herbal medicinal industries and could be used as an ingredient in ionone flavors, botanical insecticides, food spices, and personal-care products.

The dried berry of *Piper cubeba* (Piperaceae), known as the ‘cubeb pepper’ or ‘tailed pepper’, have been widely used as a popular spice, with beneficial properties, including anti-inflammatory, analgesic, anti-proliferative, and leishmanicidal activities [5,6], and a flavoring agent for gins and cigarettes consumed throughout Europe as well as in many other Polynesian countries [7].

*L. cubeba* has been described as the cubeb berry in the Chinese Pharmacopoeia, whereas *P. cubeba* has been also listed as the origin of cubeb berries. Both of the two cubeb berries have provided special flavors for daily life. It is well known that the flavor of food is tightly related to the stimulation of the human chemical senses, odor and taste; meanwhile, the odor is mainly caused by different volatile compositions [8]. Therefore, it is an appropriate method to discriminate the two ambiguous cubeb berries by the identification of volatiles.

The volatile profiles of cubeb berries were studied previously using hydro-distillation (HD) for the extraction of essential oil. Li et al. [1] investigated the inhibitory activities of *L. cubeba* essential oil and found the main chemical composition including limonene oxide, d-limonene. Hydro-distillation (HD) and simultaneous distillation/extraction (SDE) have been the common methods for the volatile extraction of different materials [9]. SDE united the advantages of liquid–liquid and steam distillation-extraction and has been widely recognized as a relative convenient extraction method for essential oils. However, distillation at elevated temperatures may lead to the loss of some compounds and generate artefacts due to thermal changes. Headspace solid-phase microextraction (HS-SPME) based on the distribution coefficient of analytes among the sample matrix, the gas phase, and the fiber coating was a simple, rapid, and solvent-free technique [10]. SDE and SPME methods have been widely used to extract the volatiles of potato products [11], milk products [12], tea [13], and meat products [14]. However, to the best of our knowledge, headspace volatiles of cubeb fruit, directly contacting human olfactory receptors and closely associated with an overall special spicy aroma, were still not analyzed until now. Rather than rate techniques (HD, SDE, and SPME) as more superior to another in performance, HD, SDE, and SPME could be regarded as the techniques that provide complementary information for each other [14]. In present study, we evaluated the volatile profiles of different cubeb berries by the use of three extraction methods (HD, SDE, and SPME) coupled to GC-MS and combined with the principal component analysis, which would provide more comprehensive data for the discrimination of ambiguous cubeb fruit.

It is known that only a small portion of the large number of volatiles in a food matrix contribute to its overall perceived odor. GC-O is an appropriate analytical solution, as the eluted substances are perceived simultaneously by two detectors, one of them being the human olfactory system. Therefore, GC-O provides not only an instrumental, but also a sensorial analysis [8]. The GC-O technique has been widely used to the identification of aroma-active compounds from different fruits [15,16,17,18]. However, so far, GC-O technique has not been applied to the identification of aroma-active compounds of cubeb fruit.

In this essay, we report our latest study of characterization of cubeb fruit, which consisted of the following steps: (a) different pretreatment methods (HD, SDE, and SPME) were applied to obtain the volatiles and essential oils of different cubeb fruits; (b) GC-MS-O were adopted to characterize aroma-active compounds of the cubeb fruits; (c) specific aromas contributed to the discrimination of *L. cubeba* and *P. cubeba* were identified by chemometrics, which would provide helpful clues for the characteristics of aroma in different cubeb fruits and provide accurate information on the authenticity of the cubeb products.

## 2. Results and Discussion

### 2.1. Optimization of SPME

**Figure 1:**
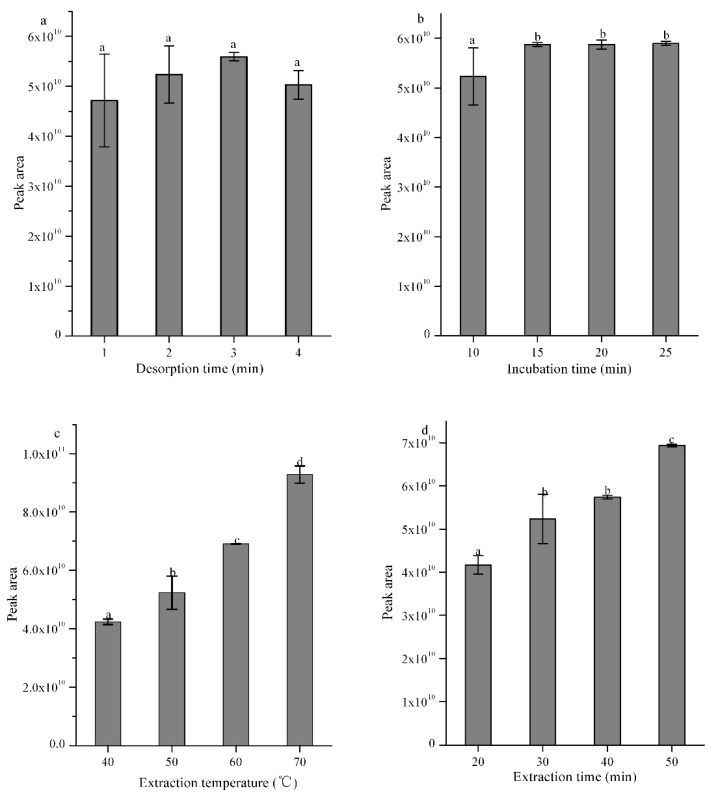
Effects of different factors—(**a**) desorption time; (**b**) incubation time; (**c**) extraction temperature; and (**d**) extraction time—on the peak areas of different volatile compounds of cubeb berries captured by DVB/CAR/PDMS. Different letters (a, b, c, and d) on the top of columns indicate significant differences (*p* < 0.05)

The volatile compounds in cubeb fruits were extracted using HS-SPME and the highest peak area response was selected in order to optimize the main parameters. These different desorption times, incubation times, extraction temperatures, and extraction times were optimized based on the total ion response in the GC-MS [19]. As shown in Figure 1a, study of desorption time including 1, 2, 3, and 4 min was tested. The peak areas of different volatiles were not significantly affected by the desorption time (*p* < 0.05). In order to clean the fiber sufficiently, 3 min was chosen as the desorption time in the present work.

Figure 1b shows the effect of incubation time (10, 15, 20, and 25 min) on the detection of total volatiles. The total volatile amounts significantly rose with increasing incubation time. However, there was no significant difference between 15, 20, and 25 min (*p* < 0.05), which indicated that a 15 min incubation time would allow distributions between the fiber, the vial headspace, and the analytes to reach an equilibrium.

Study of the extraction temperature including 40, 50, 60, and 70 °C was investigated as illustrated in Figure 1c. The peak areas of volatiles were significantly affected by the extraction temperature. In order to avoid aroma changes cause by higher temperature, 60 °C was chosen as the extraction temperature.

Similarly, Figure 1d showed that there was no significant difference between 30 min and 40 min (*p* < 0.05). In order to avoid the fiber desorption caused by the long time exposure in the vial, 30 min was chosen as the optimum extraction time.

Therefore, the optimal extraction conditions were as follows: desorption time, 1 min; incubation time, 15 min; extraction temperature, 60 °C; and extraction time, 30 min. These conditions were applied during the headspace extraction of volatile compounds from cubeb fruits.

### 2.2. Identification of Aroma Compounds

**Figure 2:**
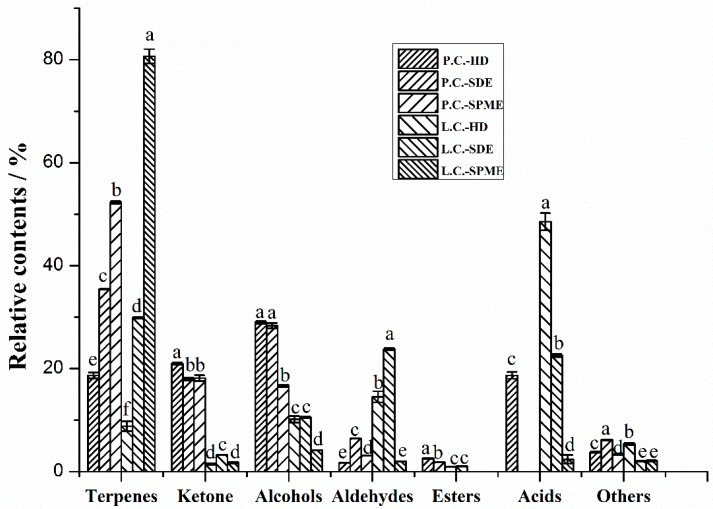
Chemical compositions of volatile compounds in different extracts (HD, SDE, and SPME) of *L. cubeba* and *P. cubeba*. Different letters (a, b, c, d, and e) indicate significant differences (*p* < 0.05).

To obtain a wider volatile profile and better discriminate the two cubeb berries, three extraction methods (HD, SDE, and SPME) were used (Table 1). In total, 90 volatile compounds were identified by HD, SDE, and SPME combined with GC-MS. Seventy-three volatile compounds belonged to different chemical families: terpenes (8.82–80.65% for *C. cubeba*; 18.69–52.27% for *P. cubeba*), ketones (1.49–3.24% for *C. cubeba*; 17.97–20.99% for *P. cubeba*), alcohols (4.16–10.56 % for *C. cubeba*; 16.68–28.97% for *P. cubeba*), aldehydes (2.02–23.79% for *C. cubeba*; 1.71–6.43% for *P. cubeba*), esters (0–1.06% for *C. cubeba*; 0.98–2.57% for *P. cubeba*), and acids (2.39–48.55% for *C. cubeba*; 0–18.69% for *P. cubeba*). In order to highlight the differences between the two cubeb fruit with different extraction techniques in a simple and immediate way, Figure 2 showed the comparison of the relative percentages of the main chemical families present in cubeb berries.

One of the most abundant chemical families identified in cubeb fruits was terpenes. To our knowledge, the literature dealing with the comparison of different extraction methods of volatile compounds in cubeb berries is scare. Wang et al. [20] studied the chemical composition of the essential oil obtained only by HD of different parts (root, stem, leaf, flower, and fruit) of *L. cubeba* and showed that citral and limonene were the main constituents. In the present work, for *L. cubeba*, d-limonene was one of the main terpenes for the SDE (10.57%) and SPME extracts (38.89%), but it was not detected in the HD extract. Wang et al. [21] reported that some other terpenes, such as α-pinene, β-pinene, and β-caryophyllene, were also detected as the main volatile compounds in the bio-oils produced from *L. cubeba* seed by hydrothermal liquefaction. α-Pinene (5.74%), β-pinene (6.22%), d-limonene (9.84%), and caryophyllene (5.57%) were also the main terpenes for the SPME extract of *P. cubeba*. γ-Terpinene (A13, 0.12–0.39%), α-copaene (A19, 0.13–1.15%), caryophyllene (A23, 5.53–9.11%), and humulene (0.77–4.33%) were the common terpenes detected in all the three different extracts of two cubeb berries. Three terpenes could only be detected in all the extracts of P. cubeb, including α-cubebene (A18, 0.4–0.52%), bicyclosesquiphellandrene (A27, 2.41–5.28%), and α-farnesene (A30, 0.13–0.16%). Cubebene was also identified in the direct analysis in real time mass spectrometry (DART-MS) fingerprint of *P. cubeba* studied by Kim et al. [7]. The extracts obtained by SPME were rich in terpenes (80.65% for *L. cubeba* and 52.27% for *P. cubeba*) in comparison with those from HD (8.82% for *L. cubeba* and 18.69% for *P. cubeba*) and SDE (29.9% for *L. cubeba* and 35.4% for *P. cubeba*). However, SDE with solvents tends to extract a higher amount of the volatile monoterpenes than SPME in bay leaf [22], French beans [23], and wines [24]. These differences may be due to the matrix effect in releasing volatile compounds as each spice had a characteristic plant tissue structure. Meanwhile, strong oxidation and degradation of terpenes may occur for HD and SDE extracts because of higher temperature and longer time [25].

For ketones, 6-methyl-5-hepten-2-one (B1) and camphor (B2) were both detected in all the cubeb berries samples. As irregular terpene, 6-methyl-5-hepten-2-one is probably derivative of carotenoids produced by enzymatic action [26]. Camphor (17.59–20.6%) was detected as the major ketones in all the extracts of *P. cubeba*. The contents of ketones in *P. cubeba* (17.97–20.99%) with different extraction methods were significantly higher than in the *L. cubeba* (1.49–3.24%) by one-way ANOVA (Figure 2).

Alcohols were also present with a high proportion in cubeb fruit, and the contents of alcohols in *P. cubeba* (16.68–28.97%) with different extraction methods were significantly higher than in the *L. cubeba* (4.16–10.56%) by a One-way ANOVA (Figure 2). Terpinen-4-ol (C7) could be detected in all the extracts of *L. cubeba* and *P. cubeba*. Linalool (C2, 14.89–21.31%) was the most abundant volatile and could be found in all the extracts of *P. cubeba*.

Citral was present as the most abundant aldehyde compound in *L. cubeba* extracts. This result is in accordance with the study by Wang et al. [20], who reported that citral was one of the main constituents in the fruit oil of *L. cubeba* extracted by HD. The contents of 3,7-dimethyl-2,6-octadienal in *L. cubeba* was higher than *P. cubeba*. Only 10 esters were identified in the current work, most were relatively high-boiling esters and with lower contents than other chemical families in cubeba berries.

Only two kinds of acids, decanoic acid and dodecanoic acid, were mainly extracted by HD and SDE with longer extraction time and higher temperature. The lack of acids with low volatility in SPME extracts may be caused by the low extraction temperature during the extraction process. Most acids may exist as esters form or have been changed to aldehydes, alcohols, or other secondary metabolites [13]. The contents of acids in *L. cubeba* (2.39–48.55%) with different extraction methods were significantly higher than in the *P. cubeba* (0–18.69%) by one-way ANOVA (Figure 2).

### 2.3. Aroma-Active Compounds by GC-MS-O

The extracts obtained by SPME were analyzed to assess the aroma-active compounds of the cubeb fruits using GC-O. Table 2 listed the identified aroma-active compounds of the *L. cubeba* and *P. cubeba*. A total of 12 compounds were tentatively found to be the aroma-active compounds at olfactometry port for odor description in GC-O analysis, including eight terpenes, one ketone, two alcohols, and one aldehyde. The odor descriptions of all the aroma-active compounds identified in the volatiles of *L. cubeba* and *P. cubeba* were basically similar to the reported of other fruits, such as blackberry [27], bayberry [17], strawberry [28], orange [29], and gooseberry [30].

According to the evaluation of the odor and the odor description of the reported, it can be concluded that the flavor of turpentine-like might be caused by α-pinene (A1), β-pinene (A4), α-phellandrene (A6), γ-terpinene (A13), and terpinen-4-ol (C7); the fruity and flower flavor might be due to the presence of D-limonene (A10), citral (D3) with higher level in *L. cubeba*, and linalool (C2) with higher level in *P. cubeba*; the herbal flavor might come from β-ocimene (A12), α-cubebene (A18) identified only in *P. cubeba*, and caryophyllene (A23); camphoraceous flavor might be caused by camphor (C2), which is stronger in *P. cubeba* with higher content than in *L. cubeba*. It is interesting to note that α-cubebene was in very low proportions, also had high detection frequencies in all the extracts of *P. cubeba*. α-cubebene had been detected in other study for cubeb fruit [7].

### 2.4. Principal Component Analysis (PCA)

**Figure 3:**
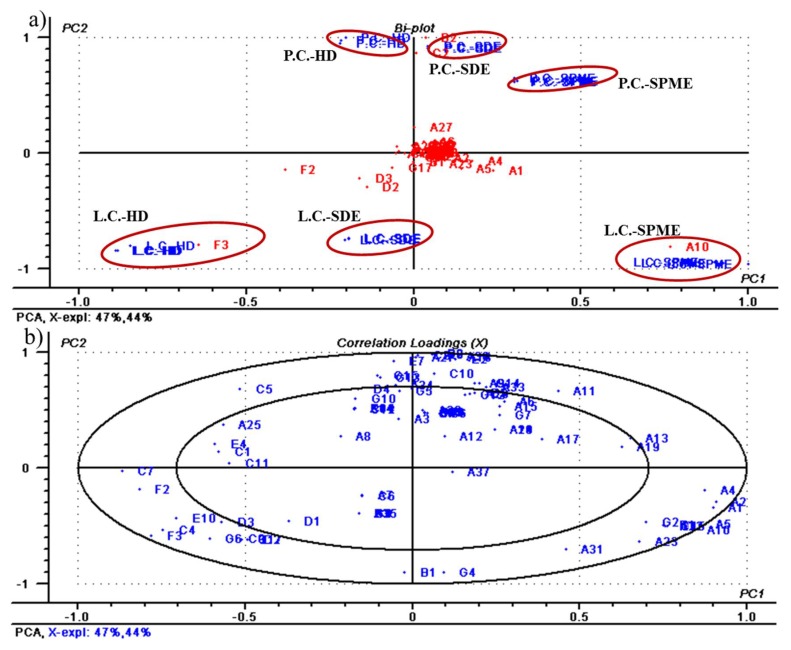
PC1 vs. PC2 scatter plot for variability among the different cubeba samples: (**a**) bio-plots of PC1 & PC2 of the volatile compounds of different extracts (HD, SDE, and SPME) of *L. cubeba* and *P. cubeba*; (**b**) relation between the volatile compounds (loadings).

Principal component analysis (PCA) is an unsupervised clustering method and could reduce the dimensionality of multivariate data and preserve most of the variance therein [31]. To get a clear distribution of the volatiles with the separation of the samples, PCA was applied to the data presented in Table 1, the first two principal components explained nearly 91% of the total variability of the GC-MS data set between the samples, is shown in Figure 3a. The corresponding loading weight plot, establishing the magnitude of each volatile component (variable), is illustrated in Figure 3b. Figure 3 plots the samples on the coordinate grid defined by the first two principal components and showed that PC1 and PC2 separated the *L. cubeba* samples from the *P. cubeba* samples.

Principal component 1 (PC1) and PC 2, explained 47% and 44% of the total variance among the sample batches, showed that the cubeb berries discrimination based on varietal volatile profile. The extracts (HD, SDE, and SPME) of *P. cubeba* was projected in positive PC2 and highly associated with α-cubebene (A18, 0.4–0.52%), bicyclosesquiphellandrene (A27, 2.41–5.28%), camphor (B2, 17.59–20.6%), and linalool (C2, 14.89–21.31%). The extracts (HD, SDE, and SPME) of *L. cubeba* were located in the negative PC 2 with high d-limonene (A10, 0–38.89%), 3,7-dimethyl-2,6-octadienal (0.72–11.32%), citral (D3, 1.09–11.85%), and dodecanoic acid (F3) content (Figure 3b).

In conclusion, 12 volatiles were identified as aroma-active compounds in both cubeb berries with mainly ‘turpentine-like’, ‘fruity and flowery’, ‘herbal’, and ‘camphoraceous’ flavors. Principal component analysis was further applied to the data of GC-MS, which differentiated and discriminated the two ambiguous cubeb berries according to their unique volatile compounds. *Litsea cubeba* was characterized by higher level of d-limonene (‘fruit, citrus’ note, 0–38.89%), 3,7-dimethyl-2,6-octadienal (0.72–11.32%), citral (‘fruit, lemon’ note, 1.09–11.85%) and dodecanoic acid (1.89–32.9%). α-Cubebene (‘herb’ note, 0.4–0.52%) was identified as a marker compound for *Piper cubeba* with higher camphor (‘camphoraceous’ note, 17.59–20.6%), and linalool (‘flower’ note, 14.89–21.31%) contents.

## 3. Materials and Methods

### 3.1. Materials and Chemicals

*Litsea cubeba* was collected from Guizhou province (Guiyang, China) and *Piper cubeba* was from Yunnan province of China (Yuxi, China). The collected cubeb samples were kept in a dry and dark place and stored at 4 °C in order to minimize any deteriorative changes to the volatile components of the cubeb berries until their processing. For the precise measurements of GC-MS-O (Agilent Technologies Inc., Santa Clara, CA, USA), cubeb fruit samples were ground to a fine powder using a grinder.

The *n*-alkane standard (C8–C20) was provided by Sigma-Aldrich Chemical Co. (Sigma Chemical Co., St. Louis, MO, USA).

### 3.2. Hydro-Distillation (HD)

All the air-dried cubeb berries samples (an amount of 100 g each) were subjected to hydro-distillation using a Clevenger-type apparatus to extract essential oil using the reported methods with some modifications [20,32]. The Clevenger-type apparatus consisted of a 2000 mL glass flask, a vertical tube, a condenser, a measuring tube with stopcock, and a return tube. The return tube connected the bottom of the measuring tube to the vertical tube, which combined with the top of the condenser. The flask was filled with 1200 mL of distilled water and heated by an electric heating mantle. The extraction time was 4 h, after which no more essential oil was obtained. The vapor mixture of water–essential oil produced in the flask passed through the condenser and then the distillate was collected. The essential oil in the upper layer of the distillate was dried over anhydrous sodium sulfate (Na_2_SO_4_) and stored at 4 °C until subsequent GC-MS analysis.

### 3.3. Simultaneous Distillation Extraction (SDE)

SDE was performed in a modified Lickens–Nickerson apparatus (Chrompack, Netherlands) [33]. A 25 g measure crushed air-dried cubeb berry, with 1.6g sodium chloride and 200 mL distilled water, was placed in a 500 mL flask. The sample and 40 mL of a mixture of pentane–diethyl ether (1:1 *v/v*) solvent placed in another flask were heated up to their boiling points and the temperature conditions were maintained for about 3 h. After cooling to ambient temperature for 10 min, the pentane-diethyl ether extract was dried over anhydrous Na_2_SO_4_. The extract was kept at 4 °C until subsequent GC-MS analysis [26].

### 3.4. Optimization of SPME Conditions

A SPME (Supelco, Inc., Bellefonte, PA, USA) fiber (50/30 µm divinylbenzene/carboxen/polydimethylsiloxane; DVB/CAR/PDMS) was used for volatile extraction after the fiber had been conditioned at 270 °C for 1 h. The ground samples were passed through a 20 mesh sieve to achieve uniform particle size. A 1.5 g measure of the sieved cubeb fruits powder was placed in a 20 mL vial with a sealed cap and equilibrated in a laboratory stirrer/hot plate (model PC-420, Corning Inc. Life Science, Acton, MA, USA). Then, a stainless steel needle, housing the SPME fiber, was placed through a hole to expose the fiber in the vial [19]. Three independent extractions were done for each cubeb fruit sample.

To improve the volatile absorption, the SPME parameters were optimized: desorption time (1, 2, 3, and 4 min); incubation time (10, 15, 20, and 25 min); extraction temperature (40, 50, 60, and 70 °C); extraction time (20, 30, 40, and 50 min). For each parameter investigated, the analysis was conducted in triplicate. 

### 3.5. Analysis of Volatiles by GC-MS

7890A gas chromatograph with 5975C mass spectrometer selective detector (Agilent Technologies Inc., Santa Clara, CA, USA) was used, and a DB-5 capillary column (30 m ×0 .25 mm × 0.25 µm) was applied for GC-MS. The extraction was injected into the inlet of GC-MS and desorbed at 250 °C for 3 min. The injection port was operated in splitless mode, helium (99.999%) was used as carrier gas at the flow rate of 1.2 mL/min. The initial oven temperature was 40 °C (2 min), ramped at 3 °C min^−1^ to 170 °C (5 min), and then ramped at 10 °C min^−1^ to 260 °C (5 min). Mass detector conditions were performed by EI (electronic impact) mode at 70 eV, source temperature at 230 °C, mass spectra acquisition range of 45–500 amu, scanning rate of 3.18 amu/s. The transfer line temperature was 280 °C. The volatile compounds were identified by comparing the mass spectra with mass-spectral library (NIST 2011), retention index (RI), aroma description, and matching against the published data [34]. Each extract was analyzed in triplicate. Mean data and relative standard deviation (mean ± SD) of volatiles were reported.

### 3.6. GC-MS-Olfactometry

GC-MS-O was performed by trained panelists on a sniffing port (Sniffer 9000, Brechbühler, Schlieren, Switzerland). The modified method described earlier by Pang et al. [18] was used in this study and the conditions of SPME and GC-MS were the same to the volatile analysis described above.

Four trained panelists take part in the detection frequency analysis (DFA) combined with GC-MS-O for identification aroma-active compounds. The panel consisted of an age from 20 to 35 years (mixed of male and female). The panelists were trained by solutions of artificial odorants and different cubeb berries samples to be familiar with the odor descriptions. In total, eight runs by GC-MS-O were conducted by four assessors (two runs for one person). The judges sniffed the effluent from the mask and recorded the time and odor characteristic of the aroma-active compounds of different cubeb berries samples. When the total detection frequencies were more than twice for the odorants perceived by two different assessors at the sniffing port, the odorants were considered potential aroma-active compounds [18,35].

### 3.7. Statistical Data Analysis 

Significant differences for the volatile constituents among the cubeb berries were determined by one-way analysis of variance (ANOVA) using a SPSS statistics (version 20.0; SPSS, Inc., Chicago, IL, USA). The column figures in the context were plotted using Origin software (version 8.5; Northampton, MA, USA). The Unscrambler v.9.7 (CAMO AS, Trondheim, Norway) software was used for the statistical analysis (PCA) on volatiles.

## 4. Conclusions

In this study, the aroma compounds of two ambiguous cubeb berries were isolated by HD, SDE, and SPME pretreatment methods in order to fully obtain the complex aroma profiles of the cubeb berries and were analyzed by GC-MS-O combined with PCA. By GC-MS-O analysis, a total of 12 aroma-active compounds were found to play a key role in the characteristic flavor of the cubeb berries. The PCA results clearly indicated that the two ambiguous cubeb berries could be discriminated by the aroma profiles. *Litsea cubeba* was characterized by higher level of d-limonene, citral and dodecanoic acid; *Piper cubeba* was marked with α-cubebene, higher camphor, and linalool. Therefore, using the volatile profile combined with PCA is an appropriate method to discriminate the cubeb berries and assure the related product quality.

## Figures and Tables

**Table 1:** Relative concentrations of volatile compounds identified in cubeb fruits by different preparation methods (HD, SDE, SPME).

Codes	RI ^1^	Volatile Compounds	Chemical Formula	Concentration for *Litsea cubeba* (%) ^2^			Concentration for *Piper cubeba* (%)		
				HD ^3^	SDE ^3^	SPME ^3^	HD ^3^	SDE ^3^	SPME ^3^
Terpenes									
A1	925	α-Pinene	C_10_H_16_	nd ^4^	1.44 ± 0.01	10.81 ± 0.36	0.09 ± 0.01	1.06 ± 0.02	5.74 ± 0.11
A2	944	Camphene	C_10_H_16_	nd	0.52 ± 0	3.37 ± 0.16	0.07 ± 0	0.47 ± 0.01	2.09 ± 0.03
A3	971	β-Phellandrene	C_10_H_16_	nd	0.3 ± 0	nd	0.11 ± 0	0.93 ± 0.02	nd
A4	973	β-Pinene	C_10_H_16_	nd	1.22 ± 0.01	7.33 ± 0.13	0.21 ± 0.01	1.02 ± 0.01	6.22 ± 0.11
A5	991	β-Myrcene	C_10_H_16_	nd	1.45 ± 0.02	6.99 ± 0.42	0.16 ± 0.03	0.45 ± 0.01	1.97 ± 0.04
A6	1002	α-Phellandrene	C_10_H_16_	nd	nd	0.13 ± 0.07	0.46 ± 0.01	1.75 ± 0.02	4.55 ± 0.07
A7	1008	3-Carene	C_10_H_16_	nd	0.23 ± 0	nd	nd	0.08 ± 0	nd
A8	1015	2-Carene	C_10_H_16_	nd	0.11 ± 0	nd	0.07 ± 0	0.09 ± 0	nd
A9	1023	Cymene	C_10_H_16_	nd	0.14 ± 0	nd	0.24 ± 0.02	0.39 ± 0.01	0.7 ± 0.02
A10	1027	d-Limonene	C_10_H_16_	nd	10.57 ± 0.09	38.89 ± 1.67	nd	1.9 ± 0.03	9.84 ± 0.09
A11	1038	trans-.beta.-Ocimene	C_10_H_16_	nd	nd	0.16 ± 0.02	0.16 ± 0	0.41 ± 0.01	0.64 ± 0.02
A12	1048	β-Ocimene	C_10_H_16_	nd	4.62 ± 0.04	0.1 ± 0.01	1 ± 0.03	2.5 ± 0.01	5.35 ± 0.04
A13	1057	γ-Terpinene	C_10_H_16_	0.12 ± 0.02	0.25 ± 0	0.26 ± 0.01	0.17 ± 0.01	0.29 ± 0.01	0.39 ± 0.01
A14	1070	4-Carene	C_10_H_16_	nd	nd	nd	0.15±0.01	nd	nd
A15	1087	Terpinolene	C_10_H_16_	nd	nd	nd	nd	0.27 ± 0.01	0.63 ± 0.01
A16	1130	2,4,6-Octatriene, 2,6-dimethyl-	C_10_H_16_	nd	nd	nd	nd	nd	0.5 ± 0.02
A17	1255	3-Carene	C_10_H_16_	nd	nd	0.14 ± 0.01	nd	nd	0.79 ± 0.03
A18	1349	α-Cubebene	C_15_H_24_	nd	nd	nd	0.4 ± 0.01	0.43 ± 0.02	0.52 ± 0.02
A19	1375	α-Copaene	C_15_H_24_	0.13 ± 0.01	1.07 ± 0.12	1.09 ± 0.65	0.75 ± 0.01	1.15 ± 0.02	0.95 ± 0.02
A20	1385	(+)-3-Carene	C_10_H_16_	nd	nd	nd	nd	nd	0.33 ± 0.02
A21	1389	Bicyclosesquiphellandrene	C_15_H_24_	nd	nd	nd	nd	0.4 ± 0.01	nd
A22	1392	β-Elemene	C_15_H_24_	nd	nd	1.16 ± 0.13	nd	nd	nd
A23	1419	β-Caryophyllene	C_15_H_24_	5.53 ± 0.23	6.41 ± 0.06	9.11 ± 0.37	5.84 ± 0.06	5.58 ± 0.02	5.57 ± 0.1
A24	1438	α-Guaiene	C_15_H_24_	nd	nd	nd	nd	nd	0.16 ± 0.01
A25	1453	Humulene	C_15_H_24_	3.04 ± 0.98	0.77 ± 0.01	0.88 ± 0.06	4.33 ± 0.68	1.76 ± 0.01	1.43 ± 0.03
A26	1481	β-Copaene	C_15_H_24_	nd	nd	nd	0.08 ± 0	2.74 ± 0.01	nd
A27	1497	Bicyclogermacrene	C_15_H_24_	nd	nd	nd	4.15 ± 0.04	5.28 ± 0.07	2.41 ± 0.07
A28	1500	α-Muurolene	C_15_H_24_	nd	nd	nd	nd	0.22 ± 0.01	0.13 ± 0.01
A29	1506	Cedrene	C_15_H_24_	nd	nd	nd	nd	nd	0.1 ± 0
A30	1509	α-Farnesene	C_15_H_24_	nd	nd	nd	0.13 ± 0	0.16 ± 0.01	0.16 ± 0.01
A31	1516	α-selinene	C_15_H_24_	nd	0.11 ± 0	0.11 ± 0.01	nd	nd	nd
A32	1575	γ-Muurolene	C_15_H_24_	nd	nd	nd	nd	1.09 ± 0.03	nd
A33	1582	Alloaromadendrene	C_15_H_24_	nd	nd	0.03 ± 0	0.08 ± 0.03	0.65 ± 0.07	0.51 ± 0.12
A34	1644	copaene	C_15_H_24_	nd	nd	nd	0.06 ± 0.01	0.12 ± 0	nd
A35	1652	β-Panasinsene	C_15_H_24_	nd	0.14 ± 0	nd	nd	nd	nd
A36	1722	β-Bisabolene	C_15_H_24_	nd	nd	0.05 ± 0.01	nd	4.13 ± 0.04	nd
A37	1849	β-Farnesene	C_15_H_24_	nd	0.55 ± 0.02	0.04 ± 0.01	nd	0.04 ± 0	0.59 ± 0.07
Ketones									
B1	987	6-Methyl-5-hepten-2-one	C_8_H_14_O	1.2 ± 0.13	2.01 ± 0.02	1.55 ± 0.08	0.39 ± 0.04	0.33 ± 0.01	0.13 ± 0.01
B2	1145	Camphor	C_10_H_16_O	0.28 ± 0.03	0.22 ± 0.01	0.23 ± 0.09	20.6 ± 0.3	17.59 ± 0.4	18.03 ± 0.67
B3	1183	Pulegone	C_10_H_16_O	nd	1 ± 0.01	nd	nd	nd	nd
B4	1823	Isoshyobunone	C_15_H_24_O	nd	nd	nd	nd	0.04 ± 0.01	nd
Alcohols									
C1	1029	Eucalyptol	C_10_H_18_O	1.43 ± 0.15	2.99 ± 0.03	nd	2.17 ± 0.02	2.53 ± 0.02	nd
C2	1100	Linalool	C_10_H_18_O	3.74 ± 0.37	nd	3.17 ± 0.04	21.31 ± 0.28	18.57 ± 0.18	14.89 ± 0.17
C3	1107	1,5,7-Octatrien-3-ol, 3,7-dimethyl	C_10_H_16_O	nd	nd	nd	nd	0.21 ± 0.01	nd
C4	1143	Isopulegol	C_10_H_18_O	0.1 ± 0.01	0.11 ± 0.01	nd	0.04 ± 0	nd	nd
C5	1164	endo-Borneol	C_10_H_18_O	0.3±0.03	0.33±0.01	0.07 ± 0	0.6 ± 0.01	0.54 ± 0.01	0.24 ±0.14
C6	1165	Verbenol	C_10_H_16_O	nd	0.86 ± 0.01	nd	nd	0.29 ± 0.01	nd
C7	1190	Terpinen-4-ol	C_10_H_18_O	2.98 ± 0.12	3.19 ± 0.02	0.79 ± 0.05	2.64 ± 0.05	2.64 ± 0.02	1.14 ± 0.02
C8	1218	Carveol	C_10_H_16_O	nd	0.3 ± 0.03	nd	nd	nd	nd
C9	1228	2,6-Octadien-1-ol, 3,7-dimethyl-	C_10_H_18_O	0.63 ± 0.06	1.12 ± 0.06	nd	nd	nd	nd
C10	1229	Citronellol	C_10_H_20_O	nd	nd	nd	0.41 ± 0.1	1.12 ± 0.02	0.4 ± 0.02
C11	1256	Geraniol	C_10_H_18_O	0.99 ± 0.06	1.66 ± 0.05	nd	0.71 ± 0.01	1.76 ± 0.57	nd
C12	1610	.tau.-Cadinol	C_15_H_26_O	nd	nd	nd	0.49 ± 0.05	nd	nd
C13	1640	Muurolol	C_15_H_26_O	nd	nd	nd	nd	0.71 ± 0.01	nd
C14	1648	α-Cadinol	C_15_H_26_O	nd	nd	nd	0.6 ± 0.03	nd	nd
C15	1655	Nerolidol	C_15_H_26_O	nd	nd	0.14 ± 0.02	nd	nd	nd
Aldehydes									
D1	1154	Citronellal	C_10_H_18_O	0.48 ± 0.06	0.62 ± 0.01	0.21 ± 0.01	nd	0.46 ± 0.01	0.29 ± 0.01
D2	1241	2,6-Octadienal, 3,7-dimethyl-, (Z)	C_10_H_16_O	6.09 ± 0.54	11.32 ± 0.13	0.72 ± 0.04	0.52 ± 0.04	nd	1.1 ± 0.03
D3	1272	Citral	C_10_H_16_O	7.96 ± 0.72	11.85 ± 0.12	1.09 ± 0.03	0.62 ± 0.02	5.77 ± 0.03	1.74 ± 0.04
D4	1740	2,6,10-Dodecatrienal, 3,7,11-trimethyl-	C_15_H_24_O	nd	nd	nd	0.58 ± 0.02	0.2 ± 0.01	nd
Esters									
E1	1193	Methyl salicylate	C_8_H_8_O_3_	nd	nd	0.04±0.01	nd	nd	nd
E2	1213	Acetic acid, octyl ester	C_10_H_20_O_2_	nd	nd	nd	0.12 ± 0.01	0.17 ± 0.01	0.17 ± 0.01
E3	1285	Bornyl acetate	C_12_H_20_O_2_	nd	nd	nd	0.63 ± 0.1	0.52 ± 0.01	0.55 ± 0.01
E4	1297	Decanoic acid, methyl ester	C_11_H_22_O_2_	0.34 ± 0.02	nd	nd	0.55 ± 0.03	nd	nd
E5	1324	2,6-Octadienoic acid, 3,7-dimethyl-, methyl ester	C_11_H_18_O_2_	nd	nd	nd	nd	0.17 ± 0.01	0.11 ± 0.01
E6	1370	2,6-Octadien-1-ol, 3,7-dimethyl-acetate, (Z)-	C_12_H_20_O_2_	nd	nd	nd	0.07 ± 0	nd	nd
E7	1381	2-Propenoic acid, 3-phenyl-, methyl ester	C_10_H_10_O_2_	nd	nd	nd	0.42 ± 0.01	0.33 ± 0	0.14 ± 0.01
E8	1385	lavandulyl acetate	C_12_H_20_O_2_	nd	nd	nd	nd	0.64 ± 0.01	nd
E9	1387	Geranyl acetate	C_12_H_20_O_2_	nd	nd	nd	0.78 ± 0.02	nd	nd
E10	1661	Dodecanoic acid, methyl ester	C_12_H_26_O_2_	0.71 ± 0.05	nd	nd	nd	nd	nd
Acids									
F1	1360	Geranic acid	C_10_H_16_O_2_	nd	nd	0.17 ± 0.02	nd	nd	nd
F2	1391	n-Decanoic acid	C_10_H_20_O_2_	15.64 ± 1.51	9.29 ± 0.45	0.34 ± 0.02	14.85 ± 0.6	nd	nd
F3	1582	Dodecanoic acid	C_12_H_24_O_2_	32.9 ± 0.6	13.27 ± 0.36	1.89 ± 1.03	3.85 ± 0.31	nd	nd
Others									
G1	1053	Cyclopentene, 1-methyl-	C_6_H_10_	nd	0.13 ± 0.01	nd	nd	nd	nd
G2	1197	Estragole	C_10_H_12_O	nd	nd	0.04 ± 0.02	nd	nd	nd
G3	1223	Cyclohexene, 3,3,5-trimethyl-	C_9_H_16_	nd	0.73 ± 0.03	nd	nd	nd	nd
G4	1285	Anethole	C_10_H_12_O	0.34 ± 0.03	0.77 ± 0.01	0.6 ± 0.02	nd	nd	nd
G5	1287	Safrole	C_10_H_10_O_2_	nd	nd	nd	0.1 ± 0.09	0.23 ± 0.01	nd
G6	1326	Eugenol	C_10_H_12_O_2_	0.12 ± 0.01	0.15 ± 0.07	nd	nd	nd	nd
G7	1336	1,5,5-Trimethyl-6-methylene-cyclohexene	C_10_H_16_	nd	nd	nd	nd	0.51±0.06	2.04 ± 0.05
G8	1354	2,6-Octadiene, 2,6-dimethyl-	C_10_H_18_	nd	nd	nd	0.38 ± 0	0.43 ± 0.03	0.32 ± 0.01
G9	1392	Cyclohexane, 1-ethenyl-1-methyl-2,4-bis(1-methylethenyl)-	C_15_H_24_	nd	nd	nd	nd	0.44 ± 0.01	nd
G10	1405	Methyleugenol	C_11_H_14_O_2_	nd	nd	nd	0.71 ± 0.03	0.11 ± 0	nd
G11	1439	Ethanone, 1-(2-hydroxy-4-methoxyphenyl)-	C_9_H_10_O_3_	nd	nd	nd	nd	0.17 ± 0.01	nd
G12	1448	trans-Isoeugenol	C_10_H_12_O_2_	nd	nd	nd	nd	0.23 ± 0.01	0.11 ± 0.01
G13	1485	Naphthalene, decahydro-4a-methyl-1-methylene-7-(1-methylethenyl)-	C_15_H_24_	nd	nd	nd	0.69 ± 0.01	0.79 ± 0.01	nd
G14	1513	Naphthalene, 1,2,4*a*,5,6,8*a*-hexahydro-4,7-dimethyl-1-(1-methylethyl)-	C_15_H_24_	nd	nd	nd	0.31 ± 0	0.3 ± 0	0.81 ± 0.03
G15	1523	Naphthalene, 1,2,3,5,6,8*a*-hexahydro-4,7-dimethyl-1-(1-methylethyl)-	C_15_H_24_	nd	nd	nd	1.59 ± 0.02	1.58 ± 0.01	0.06 ± 0.01
G16	1652	Naphthalene, 1,2,4*a*,5,6,8*a*-hexahydro-4,7-dimethyl-1-(1-methylethyl)-	C_15_H_24_	nd	nd	nd	nd	1.4 ± 0.01	nd
G17	1669	Caryophyllene oxide	C_15_H_24_O	4.93 ± 0.27	0.25 ± 0.01	1.47 ± 0.09	nd	nd	nd

^1^ RI: retention indices. ^2^ %: relative concentration was expressed by peak area, and data listed were the mean of three assays ± SD (standard deviation). ^3^ HD: hydrodistillation; SDE: simultaneous distillation and extraction; SPME: solid phase micro-extraction. ^4^ nd: not detected.

**Table 2:** Principal aroma-active components of cubeb berries determined by SPME-GC-MS-O using DFA.

Codes	Odor	DF ^a^	
		*Litsea cubeba*	*Piper cubeba*
A1	pine, turpentine	4	2
A4	pine, resin, turpentine	4	6
A6	turpentine, mint, spice	0	2
A10	lemon, citrus, mint	6	6
A12	herb	4	6
A13	turpentine	4	4
A18	herb	0	4
A23	wood, herb	2	2
B2	camphor wood	2	6
C2	flower, lavender	2	8
C8	turpentine, nutmeg	4	4
D3	lemon, fruit	6	4

^a^ Sum of times detected by four assessors.
